# Gastric adenocarcinoma of fundic gland mucosa type localized in the submucosa: A case report

**DOI:** 10.1097/MD.0000000000012341

**Published:** 2018-09-14

**Authors:** Atsushi Uchida, Masayoshi Ozawa, Yumi Ueda, Yoko Murai, Yuka Nishimura, Hiromi Ishimatsu, Yoshimi Okouchi, Kazuya Ishiguro, Yoshitaka Hamada, Rumiko Sasamoto, Masashi Watanabe, Naoki Sano, Ryoichi Miyamoto, Satoshi Inagawa, Kazunori Kikuchi

**Affiliations:** a Department of Pathology; b Department of Laboratory; c Department of Digestive Endoscopy; d Department of Gastroenterological Surgery, Tsukuba Medical Center Hospital, Tsukuba, Ibaraki, Japan.

**Keywords:** endoscopic submucosal dissection, endoscopic ultrasound-guided fine-needle aspiration, gastric adenocarcinoma of fundic gland mucosa type, gastric adenocarcinoma of fundic gland type, gastric submucosal tumor, heterotopic gastric glands

## Abstract

**Rationale:**

Gastric adenocarcinoma of fundic gland type (GA-FG) is a new histological type of gastric cancer manifesting with differentiation into a fundic gland. Furthermore, gastric adenocarcinoma of fundic gland mucosa type (GA-FGM) is a tumor that shows differentiation into not only a fundic gland but also foveolar epithelium and a mucous gland. These tumors tend to invade the submucosal layer. However, no cases of these tumors being localized only in the submucosa have been reported. Here, we present a case of GA-FGM localized in the submucosa and describe the cytological features of this tumor. To our knowledge, this is the first reported case of GA-FGM localized in the submucosa.

**Patient concerns:**

A man in his early 70s was referred to our institution because of the detection of a gastric submucosal tumor during a health checkup.

**Diagnoses:**

Gastric adenocarcinoma of fundic gland mucosa type.

**Interventions:**

Endoscopic ultrasound-guided fine-needle aspiration (FNA), endoscopic submucosal dissection (ESD), and total gastrectomy with lymph node dissection were performed.

**Outcomes:**

The FNA specimen showed epithelial cells with low-grade atypia. In the ESD specimen, adenocarcinoma showing a gastric fundic gland mucosa-like morphology was observed. Immunohistochemical analysis showed positive staining for pepsinogen I, H+/K+-adenosine triphosphatase, MUC6, and MUC5AC and negative staining for MUC2 and CD10, indicating tumor differentiation into fundic gland mucosa. Therefore, the tumor was diagnosed as GA-FGM, with localization in the submucosal layer. Total gastrectomy and lymph node dissection were performed because of the positive margins of the ESD specimen. Neither residual tumor nor lymph node metastasis was detected; however, many foci of heterotopic gastric glands (HGGs) were observed in the gastric wall, suggesting that GA-FGM arose from an HGG. After treatment, no recurrence was observed during a 1-year follow-up period.

**Lessons:**

Various tumors may arise from HGGs. Furthermore, when an FNA specimen shows gastric fundic gland mucosa-like epithelial cells with weak atypia, the possibility of GA-FG and GA-FGM should be considered.

## 1 Introduction

Gastric adenocarcinoma of fundic gland type (GA-FG) is a new entity of low-grade, well-differentiated gastric adenocarcinomas proposed recently by Ueyama et al.^[1]^ It is defined as a tumor that shows differentiation into a gastric fundic gland and shows positive immunohistochemical staining with pepsinogen I (the marker of chief cells) or with H+/K+-adenosine triphosphatase (ATPase) (the marker of parietal cells).^[1]^ GA-FG is a rare tumor and accounts for 1.6% of all gastric carcinomas.^[2]^ In addition to the differentiation into the fundic gland, GA-FG with differentiation into foveolar epithelium and a mucous gland has also been reported, and it is referred to as gastric adenocarcinoma of fundic gland mucosa type (GA-FGM).^[3]^ So far, only 9 cases of GA-FGM have been reported.^[3–5]^ GA-FG typically shows an elevated lesion similar to a submucosal tumor, and it often infiltrates into the submucosal layer and has a small diameter.^[6]^ According to most of the previous reports on GA-FGM, this tumor shows submucosal invasion.^[5]^ However, no report has mentioned localization of this tumor in the submucosal layer. Furthermore, no report has presented the cytological features of this tumor. Here, we present a case of GA-FGM localized in the submucosa and describe the cytological features of this tumor. To our knowledge, this is the first reported case of GA-FGM localized in the submucosa.

## 2 Case presentation

**Figure 1:**
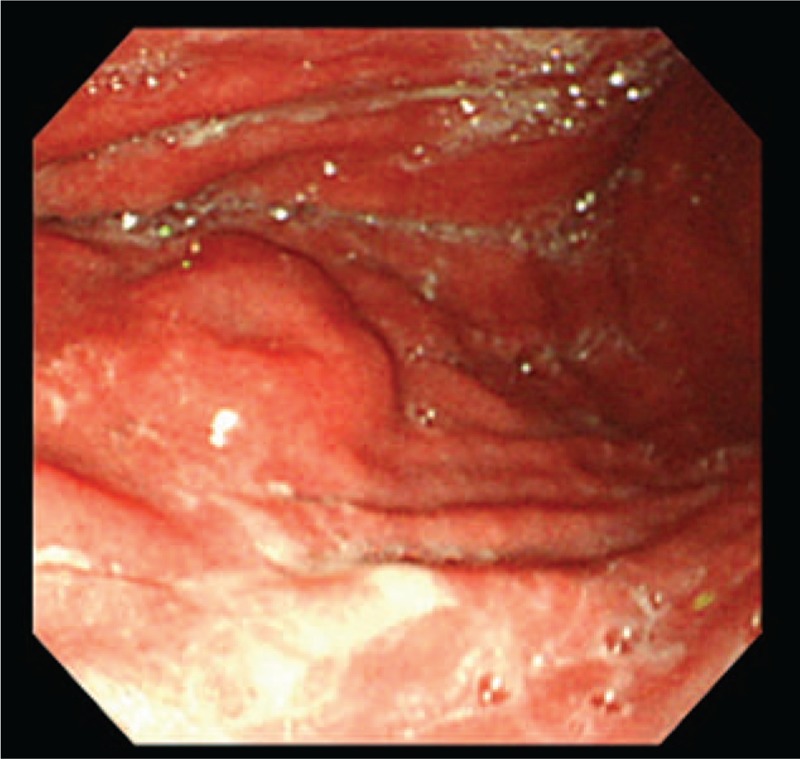
Endoscopic findings. A submucosal tumor is observed.

**Figure 2:**
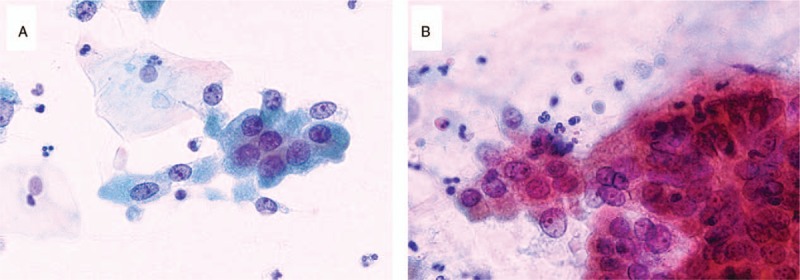
Fine-needle aspiration cytology findings. Many epithelial cells with mild atypia are observed. (A) Epithelial cells showing granular cytoplasm that stained light green (Pap. staining, ×40). (B) Epithelial cells showing abundant mucus (Pap. staining, ×40).

**Figure 3:**
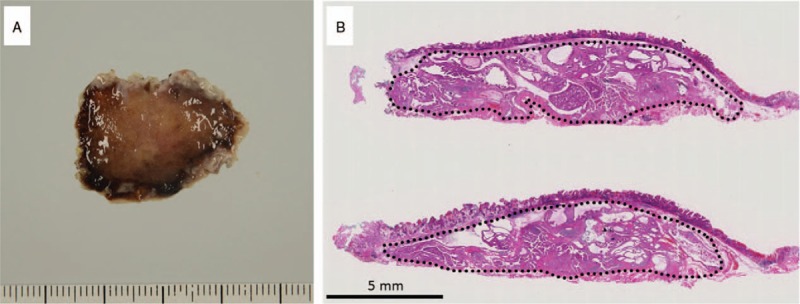
(A) Macroscopic findings. The endoscopic submucosal dissection-resected specimen showing no tumor exposed to the mucosal surface. (B) Histological findings. The black line indicates gastric adenocarcinoma of fundic gland mucosa type, which is observed only in the submucosa.

**Figure 4:**
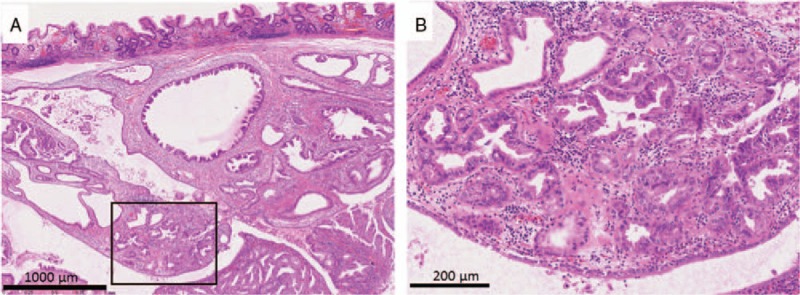
Histological findings. Low-atypical cells, similar to the cells of the gastric foveolar epithelium, mucinous gland, or fundic gland, showing proliferation and forming large and small irregular glands in the submucosa. The square in the photograph shows a magnified section.

**Figure 5:**
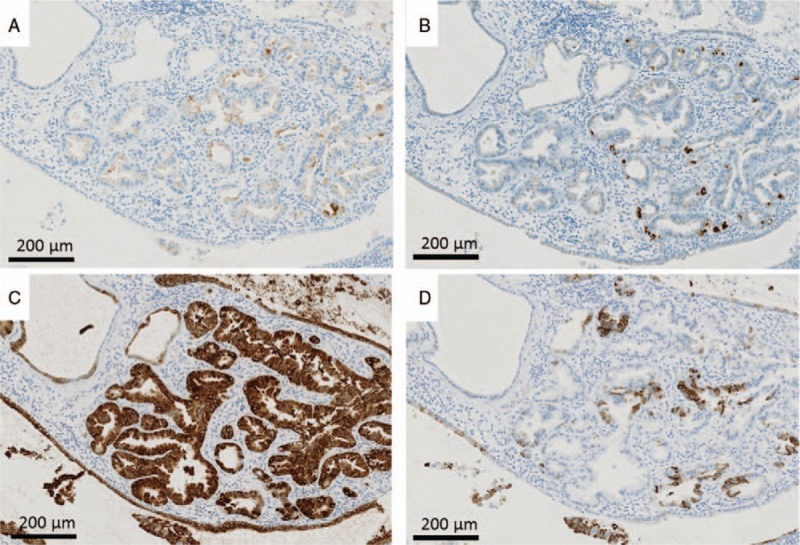
Immunohistochemical findings. The tumor shows scattered positivity for pepsinogen I (A) and H+/K+-adenosine triphosphatase (B), strong positivity for MUC6 (C), and partial positivity for MUC5AC (D).

A man in his early 70s underwent upper endoscopic examination during a health checkup; during the examination, a submucosal tumor measuring 20 mm was detected in the greater curvature of the middle body of the stomach (Fig. 1). Following this, the patient visited our hospital, and endoscopic ultrasound-guided fine-needle aspiration (FNA) of the lesion was performed. Cytology revealed many epithelial cells showing sheet-like clusters or mildly overlapping clusters and isolated scattered cells in a background of inflammatory cells and mucin. The cytoplasm of the epithelial cells contained granules that stained light green or had abundant mucus (Fig. 2). Because a mild increase in the nuclear chromatin and a clear nucleolus were observed, the possibility of a proliferative lesion was considered; however, it was difficult to confirm malignancy because the cells did not show distinctive atypia. Thus, endoscopic submucosal dissection (ESD) was performed. On assessment of the ESD specimen, a tumor measuring 23 × 15 mm was observed only in the submucosal layer (Fig. 3). Histology revealed that atypical cells, which showed a morphology similar to that of foveolar epithelium, mucous gland, and fundic gland cells, proliferated and formed large and small irregular glands (Fig. 4). No tumor or scar was detected in the lamina propria above the tumor. Immunohistochemically, the tumor showed scattered positivity for pepsinogen I and H+/K+-ATPase and strong positivity for MUC6. These findings indicated tumor differentiation into a gastric fundic gland (Fig. 5A–C). Moreover, the tumor showed positivity for MUC5AC, indicating its differentiation into gastric foveolar epithelium (Fig. 5D). Staining for MUC2, CD10, and chromogranin A was negative. The tumor showed focal and weak positivity for p53, and the Ki-67 labeling index was 14%. On the basis of the above findings, the tumor was diagnosed as GA-FGM.

**Figure 6:**
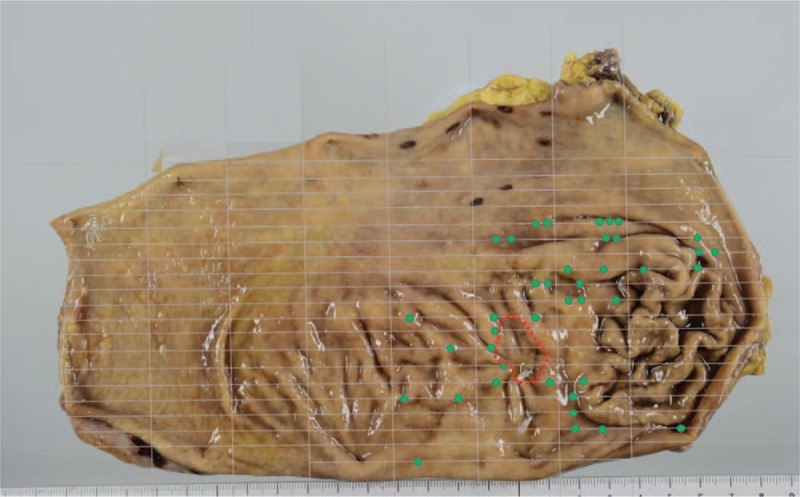
Macroscopic findings of the total gastrectomy specimen. Green dots indicate heterotopic gastric glands, and the red line indicates a scar due to endoscopic submucosal dissection.

**Figure 7:**
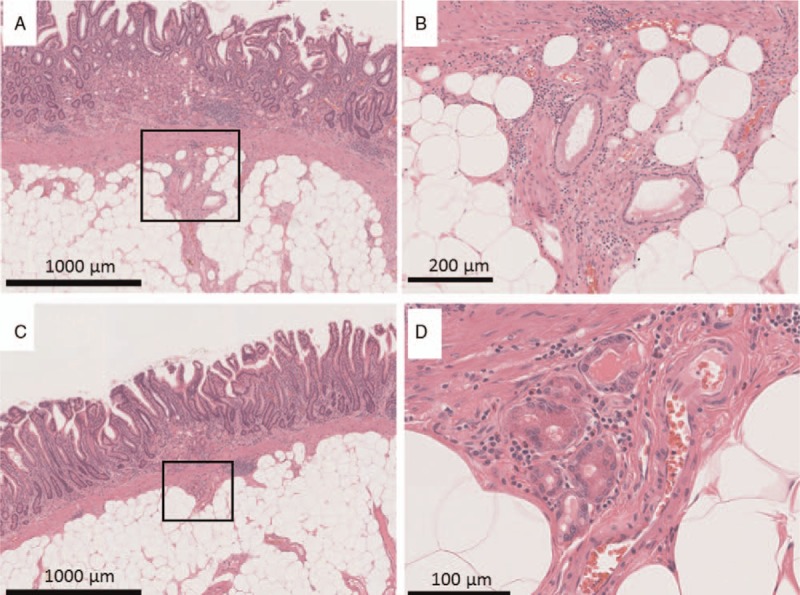
Histological findings of heterotopic gastric glands (HGGs). (A, B) HGGs showing gastric foveolar epithelium-like features and (C, D) fundic gland-like features. The square in the photograph shows a magnified section.

Because the margins of the ESD specimen were positive, total gastrectomy and lymph node dissection were additionally performed. No residual tumor or lymph node metastasis was observed; however, numerous heterotopic gastric glands (HGGs) were observed in the stomach wall (Fig. 6). In these HGGs, gastric foveolar and fundic gland- or pyloric gland-like epithelia were observed (Fig. 7). In the background of the gastric mucosa, atrophy along with intestinal metaplasia was generally observed. After treatment, no recurrence was observed during a 1-year follow-up period.

Ethical approval was not required for this case report, as it did not relate to the patient's privacy or treatment. Informed consent for the publication of this case report has been obtained.

## 3 Discussion

We encountered a patient with GA-FGM localized in the submucosa. A tumor or scar was not detected in the lamina propria above the tumor and many HGGs were observed in the submucosa. Apart from our case, only 9 cases of GF-FGM have been reported, of which 8 showed submucosal invasion and 1 was limited to the lamina propria.^[3–5]^ This indicates the tendency of this tumor to invade to submucosa, similar to FG-GM.^[6]^ However, no case was localized in the submucosa. Our case is the first to report GF-FGM localized in the submucosa, and to the best of our knowledge, GA-FG with such a growth pattern has not been reported.

There were many HGGs in the submucosa in our patient. HGGs are gastric glands observed in the submucosa, and they have been recognized as aberrant lamina propria components associated with repeated erosion and regeneration.^[7]^ Few cases of common-type gastric cancers derived from HGGs have been reported.^[8–10]^ Conversely, GA-FG has been recognized as a tumor typically arising from the normal gastric mucosa of the fundic gland region without atrophic changes or intestinal metaplasia.^[11]^ However, some cases of GA-FG arising from the gastric mucosa with atrophic changes have been reported.^[12]^ Furthermore, GA-FGs spreading to an HGG have been reported.^[13]^ Considering the above-mentioned findings, GA-FG and GA-FGM could arise from HGGs, although the transition is extremely rare. In our case, the transition of HGG to a tumor was not distinct; however, given the presence of many HCGs, no tumor in the lamina propria, and no scar, it was highly suggested that GA-FGM was derived from HGG. This indicates that a wide variety of adenocarcinomas, including rare tumors such as GA-FGM, can develop from HGGs. Thus, during the diagnosis of a GA submucosal tumor, such possibility should be taken into consideration.

In our patient, the cytological features of GA-FGM were observed. FNA performed before ESD showed epithelial cells whose cytoplasm contained granules that stained light green or had abundant mucus. Retrospectively, we believe that the former appearance indicated tumor cells showing differentiation into the fundic glands and the latter indicated tumor cells showing differentiation into the foveolar epithelium or a mucous gland. Owing to the weak atypia of the cells, it was difficult to confirm malignancy at that point. There have been no reports on the cytological features of GA-FG or GA-FGM; thus, the present report is considered to be valuable.

## 4 Conclusion

In the course of diagnosis of a gastric submucosal tumor, the possibility that various tumors may arise from HGGs should be considered. In addition, when gastric mucosa-like epithelial cells with low-grade atypia are observed, the possibility of a GA-FG or GA-FGM should be considered, although this possibility is rare.

## Acknowledgments

The authors would like to thank Dr Yao (Department of Human Pathology, Juntendo University School of Medicine) for consultation advice on the histopathological findings through pathology consultation service of the National Cancer Center, Tokyo, Japan, and the Department of Pathology, the Jikei University School of Medicine, for performing the immunohistochemical analysis with pepsinogen I and H+/K+-ATPase.

## Author contributions

**Conceptualization:** Atsushi Uchida.

**Data curation:** Masayoshi Ozawa, Yumi Ueda, Yoko Murai, Yuka Nishimura, Hiromi Ishimatsu, Yoshimi Okouchi, Kazuya Ishiguro, Yoshitaka Hamada, Rumiko Sasamoto, Masashi Watanabe, Naoki Sano, Ryoichi Miyamoto, Satoshi Inagawa.

**Supervision:** Kazunori Kikuchi.

**Writing – original draft:** Atsushi Uchida.
